# Berberine alleviates enterotoxigenic *Escherichia coli*-induced intestinal mucosal barrier function damage in a piglet model by modulation of the intestinal microbiome

**DOI:** 10.3389/fnut.2024.1494348

**Published:** 2025-01-14

**Authors:** Min Du, Xinran Liu, Xu Ji, Yue Wang, Xiaodan Liu, Chunfang Zhao, Erhui Jin, Youfang Gu, Hongyu Wang, Feng Zhang

**Affiliations:** ^1^College of Animal Science, Anhui Science and Technology University, Chuzhou, China; ^2^Anhui Province Key Laboratory of Livestock and Poultry Product Safety Engineering, Institute of Animal Science and Veterinary Medicine, Anhui Academy of Agricultural Sciences, Hefei, China; ^3^Anhui Province Key Laboratory of Animal Nutrition Regulation and Health, Chuzhou, China

**Keywords:** enterotoxigenic *Escherichia coli*, berberine, weaned piglet, intestinal mucosal barrier function, intestinal microbiome

## Abstract

**Introduction:**

Enterotoxic *Escherichia coli* (ETEC) is the main pathogen that causes diarrhea, especially in young children. This disease can lead to substantial morbidity and mortality and is a major global health concern. Managing ETEC infections is challenging owing to the increasing prevalence of antibiotic resistance. Berberine, categorized as a substance with similarities in “medicine and food,” has been used in China for hundreds of years to treat gastrointestinal disorders and bacteria-induced diarrhea. This study investigated the preventive effect of dietary berberine on the intestinal mucosal barrier induced by ETEC and the microbial community within the intestines of weaned piglets.

**Methods:**

Twenty-four piglets were randomly divided into four groups. Piglets were administered either a standard diet or a standard diet supplemented with berberine at concentrations of 0.05 and 0.1%. and orally administered ETEC or saline.

**Results:**

Dietary supplementation with berberine reduced diamine oxidase, d-lactate, and endotoxin levels in piglets infected with ETEC (*P* < 0.05). Berberine increased jejunal villus height, villus/crypt ratio, mucosal thickness (*P* < 0.05), and goblet cell numbers in the villi and crypts (*P* < 0.05). Furthermore, berberine increased the optical density of mucin 2 and the mucin 2, P-glycoprotein, and CYP3A4 mRNA expression levels (*P* < 0.05). Berberine increased the expressions of zonula occludins-1 (ZO-1), zonula occludins-2 (ZO-2), Claudin-1, Occludin, and E-cadherin in the ileum (*P* < 0.05). Moreover, berberine increased the expression of BCL2, reduced intestinal epithelial cell apoptosis (*P* < 0.05) and decreased the expression of BAX and BAK in the duodenum and jejunum, as well as that of CASP3 and CASP9 in the duodenum and ileum (*P* < 0.05). Berberine decreased the expression of IL-1β, IL-6, IL-8, TNF-α, and IFN-γ (*P* < 0.05) and elevated total volatile fatty acids, acetic acid, propionic acid, valeric acid, and isovaleric acid concentrations (*P* < 0.05). Notably, berberine enhanced the abundance of beneficial bacteria including *Enterococcus, Holdemanella, Weissella, Pediococcus, Muribaculum, Colidextribacter, Agathobacter, Roseburia, Clostridium, Fusicatenibacter*, and *Bifidobacterium*. Simultaneously, the relative abundance of harmful and pathogenic bacteria, such as *Prevotella, Paraprevotella, Corynebacterium, Catenisphaera, Streptococcus, Enterobacter*, and *Collinsella*, decreased (*P* < 0.05).

**Discussion:**

Berberine alleviated ETEC-induced intestinal mucosal barrier damage in weaned piglets models. This is associated with enhancement of the physical, chemical, and immune barrier functions of piglets by enhancing intestinal microbiota homeostasis.

## Graphical Abstract

Berberine played a preventive role against ETEC–induced intestinal mucosal barrier damage by improving the homeostasis of intestinal microbiota in piglets. 
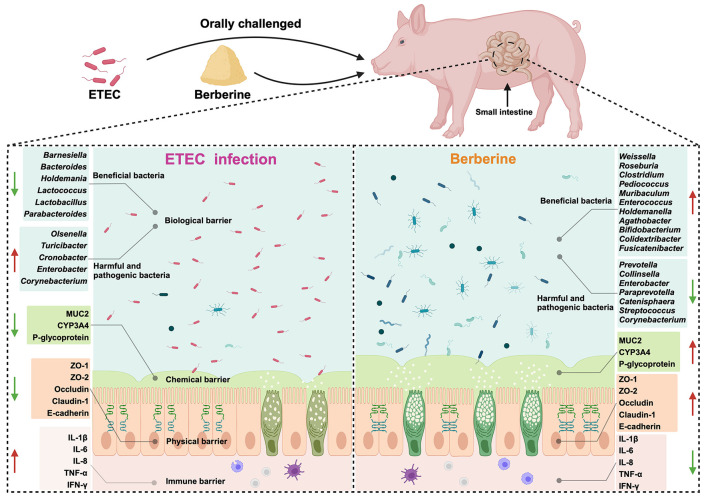

## 1 Introduction

Diarrhea is a significant global health concern. Enterotoxic *Escherichia coli* (ETEC) is a well-known pathogen that causes diarrhea, especially in children and young animals. ETEC is a common cause of traveler diarrhea in these regions (1–3). ETEC is accountable for around 220 million instances of diarrhea globally, with an estimated 75 million cases affecting children under the age of five, leading to an estimated 18,700–42,000 fatalities (2, 4).

After ETEC attaches to the intestinal mucosa, it penetrates the inner wall of the small intestine, secretes heat-stable toxins, damages the intestinal wall, and causes diarrhea (2, 5, 6). Currently, there is no licensed vaccine against ETEC-associated diarrhea (7). The management of ETEC infections through antibiotic therapy is a crucial area of study in the fields of microbiology and infectious diseases. However, research has shown that ETEC isolates exhibit significant resistance to antibiotics such as azithromycin and erythromycin (50–90%), ampicillin (85.7%), and ceftriaxone (71.4%) (3). Escalating multidrug resistance among ETEC strains diminishes the efficacy of antibiotic interventions (8). Hence, finding safe and effective alternatives to prevent ETEC infections is an urgent task and a public health concern.

Medicine and food homology (MFH) refers to substances that combine nutritional and medicinal properties and simultaneously act as nutritious foods and health-boosting herbal remedies (9). This concept has demonstrated effectiveness in regulating human health because of its abundant natural active ingredients, including polyphenols, flavonoids, polysaccharides, saponins, alkaloids, and essential oils (9, 10). Many secondary metabolites present in MFH plants have anti-inflammatory, antibacterial, and anticancer effects, indicating that MFH plants have potential applications in disease prevention and treatment (11). In recent years, the focus of medical care has shifted to a combination of prevention and treatment, leading to the incorporation of MFH plants with healing properties into diets as dietary supplements and falling within the realm of alternative therapies (12, 13).

Berberine is a natural isoquinoline alkaloid found in various MFH plants (14). Berberine has attracted considerable attention because of its wide range of pharmacological activities, including effectiveness against cancer, inflammation, and heart disease and brain protection (15). One of its outstanding features is its strong resistance to various harmful microbes, particularly gram-negative bacteria (15, 16). Berberine has been used for centuries in China to treat diarrhea and gastrointestinal disorders, particularly bacterial diarrhea (17). Despite its long history of use, there is limited research on the preventive and therapeutic effects of berberine, specifically on diarrhea caused by ETEC. Pigs are considered an ideal biomedical model for studying intestinal barrier damage and diarrhea because their physiology, metabolism, and organ size are similar to those of humans.

Therefore, in this study, we selected weaned piglets as experimental subjects to establish a diarrhea piglet model of ETEC infection. The dose of berberine was selected based on prior research demonstrating its safety in both clinical and preclinical settings (18). For instance, clinical trials have reported that berberine doses up to 1,500 mg/day are well-tolerated in humans (14, 17). Similarly, animal studies have shown that berberine exerts its pharmacological effects such as anti-inflammatory and antimicrobial effects at a maximum dose of 2,000 mg/kg and no observable adverse effects in mouse and chickens (19, 20). These findings, combined with the safety monitoring measures implemented during the study, support the safety of the berberine dose administered. This study aimed to determine the preventive effect of berberine on ETEC-induced damage to the intestinal mucosal barrier and intestinal flora of piglets, providing a potential new treatment for diarrhea caused by ETEC infection.

## 2 Materials and methods

### 2.1 Animal ethics

The animal studies were approved by the Experimental Animal Ethics Committee of the Anhui Science and Technology University (AHSTU2023006). The studies were conducted in accordance with the local legislation and institutional requirements. Written informed consent was obtained from the owners for the participation of their animals in this study.

### 2.2 Bacterial strains

The *E. coli* F4 (K88 ac) strain was purchased from the National Center for Veterinary Culture Collection of China (CVCC1500).

### 2.3 Animals, housing, and experimental design

**Figure 1:**
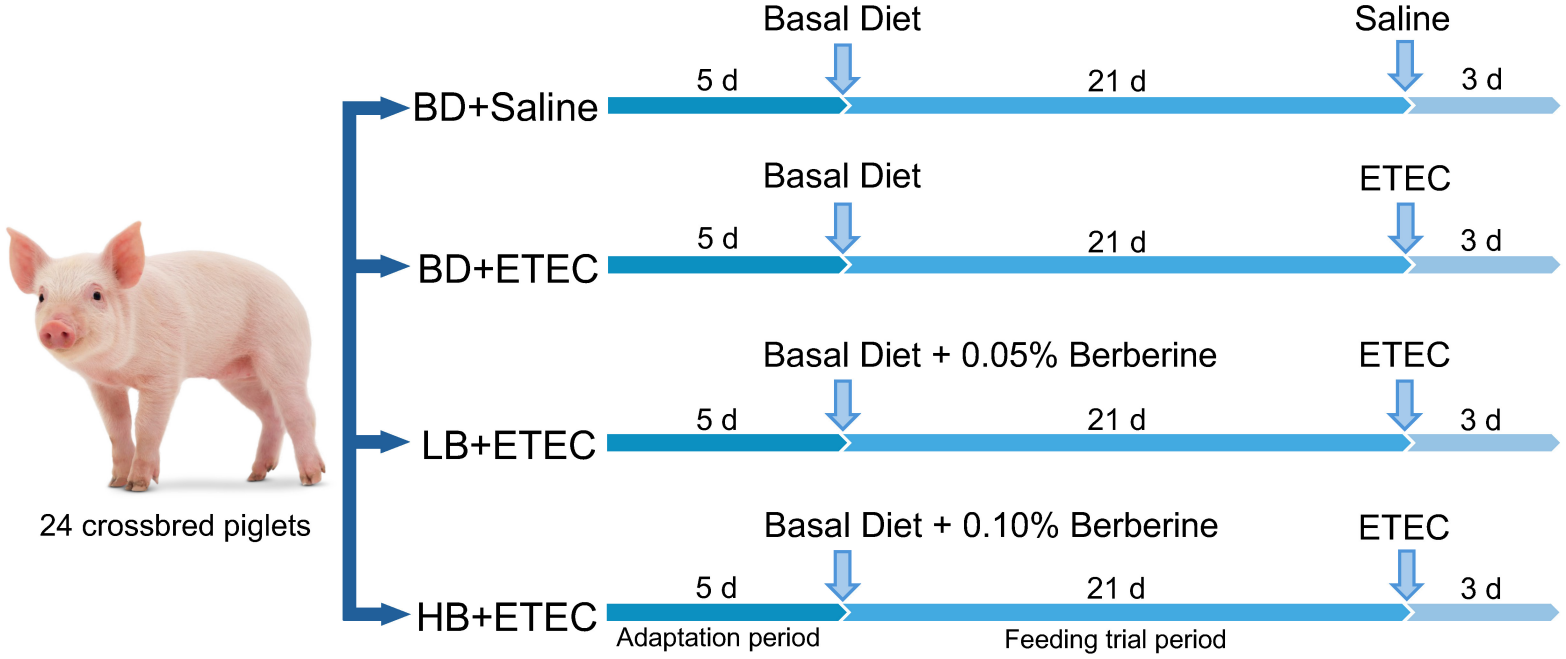
Animal experiment design. BD, basal diet; LB, basal diet with 0.05% berberine; HB, basal diet with 0.1% berberine.

For this study, 24 crossbred pigs (Duroc × Landrace × Large Yorkshire) were purchased from the local farm. Pigs were weaned at 21 days of age and placed in a controlled environment following procedures described in previous studies (21, 22). All pigs had free access to water and feed and received the same basic diet (23). The weaned piglets were randomly distributed into four experimental groups: (1) basal diet (BD) + Saline (basal diet with saline orally administered to piglets); (2) BD + ETEC (basal diet with ETEC orally administered to piglets at 10^9^ CFU per pig); (3) LB + ETEC (basal diet with 0.05% berberine, ETEC orally administered to piglets); and (4) HB + ETEC (basal diet with 0.1% berberine, ETEC orally administered to piglets). The feeding adaptation period was 5 days, and the experimental period was 21 days. At the end of the feeding period, piglets in the BD + ETEC, LB + ETEC, and HB + ETEC groups received 10 mL of ETEC (10^8^ CFU/mL) orally, whereas piglets in the BD + Saline group received 10 mL of saline for three consecutive days (Figure 1). The experimental diets were provided in meal form, and berberine chloride hydrate (purity ≥ 98%) was purchased from Aladdin Reagent Co., Ltd. (Shanghai, China) (22). For the composition and nutrients of the experimental diets, please refer to Supplementary Table S1.

### 2.4 Samples collection

On Day 25, the morning before feeding, blood was collected from the external jugular vein of the piglets. These samples were then transferred to coagulation tubes and centrifuged at 3,000 g for 10 min at 4°C. Subsequently, the serum obtained was preserved at −80°C. Piglets were euthanized humanely and dissected to collect samples of the middle parts of the duodenum, jejunum, and ileum, stored at −80°C, and prepared for RNA extraction. Moreover, some intestinal tissue was fixed with 4% paraformaldehyde. Samples from the small intestine were used for various analyses, including morphological examination, immunofluorescence staining, and a terminal deoxynucleotidyl transferase dUTP nick-end labeling (TUNEL) assay. Colonic digesta samples were gathered for volatile fatty acids (VFA) analysis and 16S rRNA gene sequencing.

### 2.5 Analysis of diamine oxidase, D-lactate, and endotoxin in plasma

Plasma diamine oxidase (DAO) activity, d-lactate, and endotoxin levels were analyzed using ELISA kits (Beijing Daktronics Technology Co., Ltd., Beijing, China) according to the manufacturer's protocol.

### 2.6 Intestinal morphology analysis

Duodenal, jejunal, and ileal sections were fixed in 4% paraformaldehyde and embedded in paraffin for histological analysis. Subsequent hematoxylin and eosin (H&E) staining was performed on the tissue samples. HE-stained sections were digitally scanned using an automated slide scanning and processing system (VM1, Motic China Group Co., Ltd., China), as described previously study (21). Villus height, crypt depth, and mucosal thickness were measured in 15 well-aligned villi per section using ImageJ 2.90 software (https://imagej.nih.gov/ij/).

### 2.7 Number of goblet cells

Alcian blue periodic acid-Schiff staining (AB-PAS) was used to detect goblet cells in the small intestine. The number of goblet cells in at least ten well-sorted villi and crypts in the duodenum, jejunum, and ileum of each piglet was determined. The results were expressed as the average number of goblet cells per 10 villi and crypts (24).

### 2.8 Immunofluorescence staining

Briefly, mucin 2 (MUC2) primary antibody (Servicebio, Wuhan, China) diluted 1:200 and Cy3-labeled goat anti-rabbit IgG secondary antibody (Servicebio) diluted 1:500 were used to detect the duodenum, jejunum, and ileum. The slides were observed using an Olympus BX63 upright fluorescence microscope, and the optical density of MUC2 was measured using imaging software (V1.18) (Olympus, Tokyo, Japan).

### 2.9 Immunohistochemistry for TUNEL

Apoptosis in small intestinal tissue was assessed using a commercially available TUNEL kit (Servicebio, Wuhan, China). Ten views were randomly obtained using a confocal laser scanning microscope (Nikon Eclipse C1, Japan) and a graphics program (Nikon DS-U3, Japan). The total number of apoptotic cells was measured using Image-Pro Plus 6.0 software. The apoptosis index (AI) was calculated using the following formula: apoptosis index (%) = number of apoptotic cells/total number of cells × 100 (21).

### 2.10 Real-time PCR

RT-PCR was performed as described previously (21). Total RNA was extracted from the duodenum, jejunum, and ileum tissues using the RNA Easy Fast Tissue/Cell Kit (TIANGEN, Beijing, China) following the guidelines provided by the manufacturer. The entire RNA sample was converted into cDNA through reverse transcription with the HiScript^®^ III 1st Strand cDNA Synthesis Kit (+gDNA eliminator) (Vazyme, Nanjing, China). Quantitative PCR was performed according to the protocols described in our previous study (21). Primers were designed using Primer 5.0 and were synthesized by Shanghai Sanggong Biotechnology Co., Ltd. (Shanghai, China). The specific primer sequences are listed in Supplementary Table S2. Since β-actin and GAPDH are sequentially expressed under different conditions, the Ct values of the target genes were normalized using the geometric mean Ct values of β-actin and GAPDH. Then the 2^−Δ*ΔCT*^ method was used to determine the relative mRNA expression of the target genes (21).

### 2.11 Determination of VFAs

We analyzed the VFAs in colonic chyme samples, including total VFA, acetic acid, propionic acid, butyric acid, valeric acid, and isobutyric acid. Fresh samples were vigorously mixed with ddH_2_O, centrifuged at 10,000 rpm for 10 min, and the supernatants were collected. The supernatant was mixed with 25% metaphosphoric acid solution, centrifuged at 20,000 g for 10 min at 4°C, and stored at 4°C for 2 h. Prior to analysis, the supernatant was filtered through a 0.45 μm polysulfide membrane. The VFA concentration (mg/g) was analyzed using an Agilent 6890 GC system (Agilent Technologies, Santa Clara, CA, USA).

### 2.12 Intestinal microbiota analysis

Intestinal microbiota analysis was performed as described previously (25, 26). Primers 341F (CCTACGGGNGGCWGCAG) and 806R (GGACTACHVGGGTATCTAAT) were used for PCR amplification of the V4-V5 regions of the bacterial 16S rRNA genes. The barcode was an 8-base sequence unique to each sample. The amplicon library was paired-end sequenced (2 × 250) on an Illumina MiSeq platform (Shanghai BIOZERON Co., Ltd.) according to standard protocols. The 16S rRNA gene sequencing data were deposited in the Sequence Read Archive (SRA) database (https://www.ncbi.nlm.nih.gov/sra) at NCBI with BioProject accession number PRJNA1110050, BioSample accession number SAMN41316931, and SRA project accession number SRR 29048606–29048629. The Chao, ACE, and Shannon diversity indices were used to assess the complexity of biological diversity.

### 2.13 Statistical analyses

After normal testing and necessary transformations, data between groups were compared using one-way ANOVA. The non-parametric Kruskal-Wallis test was used to process the relative abundance of intestinal microbial communities and data that did not follow a normal distribution. Correlation analysis was performed using Pearson's correlation test. *P* < 0.05 was considered statistically significant. All statistical analyses were performed using SPSS Statistics (version 26.0) and graphs were generated using GraphPad Prism (v10.0) software (21, 25).

## 3 Results

### 3.1 Intestinal permeability

The levels of DAO, D-lactic acid, and endotoxin in the BD + ETEC group were significantly higher than those in the BD + Saline group (*P* < 0.05). In addition, the levels of DAO, D-lactate, and endotoxin in the LB + ETEC and HB + ETEC groups were significantly lower than those in the BD + ETEC group (*P* < 0.05), but there is no significant difference compared with BD + Saline (*P* > 0.05) (Table 1).

**Table 1:** Effect of berberine on intestinal permeability of weaned piglets orally challenged with ETEC.

**Measure**	**Experimental diet**				**SEM**	***P*-value**
	**BD** + **saline**	**BD** + **ETEC**	**LB** + **ETEC**	**HB** + **ETEC**		
DAO, ng/mL	4.96^b^	6.38^a^	4.69^b^	4.31^b^	0.26	< 0.05
D-lactate, μmol/L	14.08^b^	15.74^a^	13.21^b^	13.26^b^	0.35	< 0.05
Endotoxin, EU/mL	10.21^b^	11.84^a^	10.29^b^	10.16^b^	0.22	< 0.05

^a, b^Values with different superscripts in a row indicate significant differences (*P* < 0.05).

### 3.2 Small intestinal histomorphology

**Figure 2:**
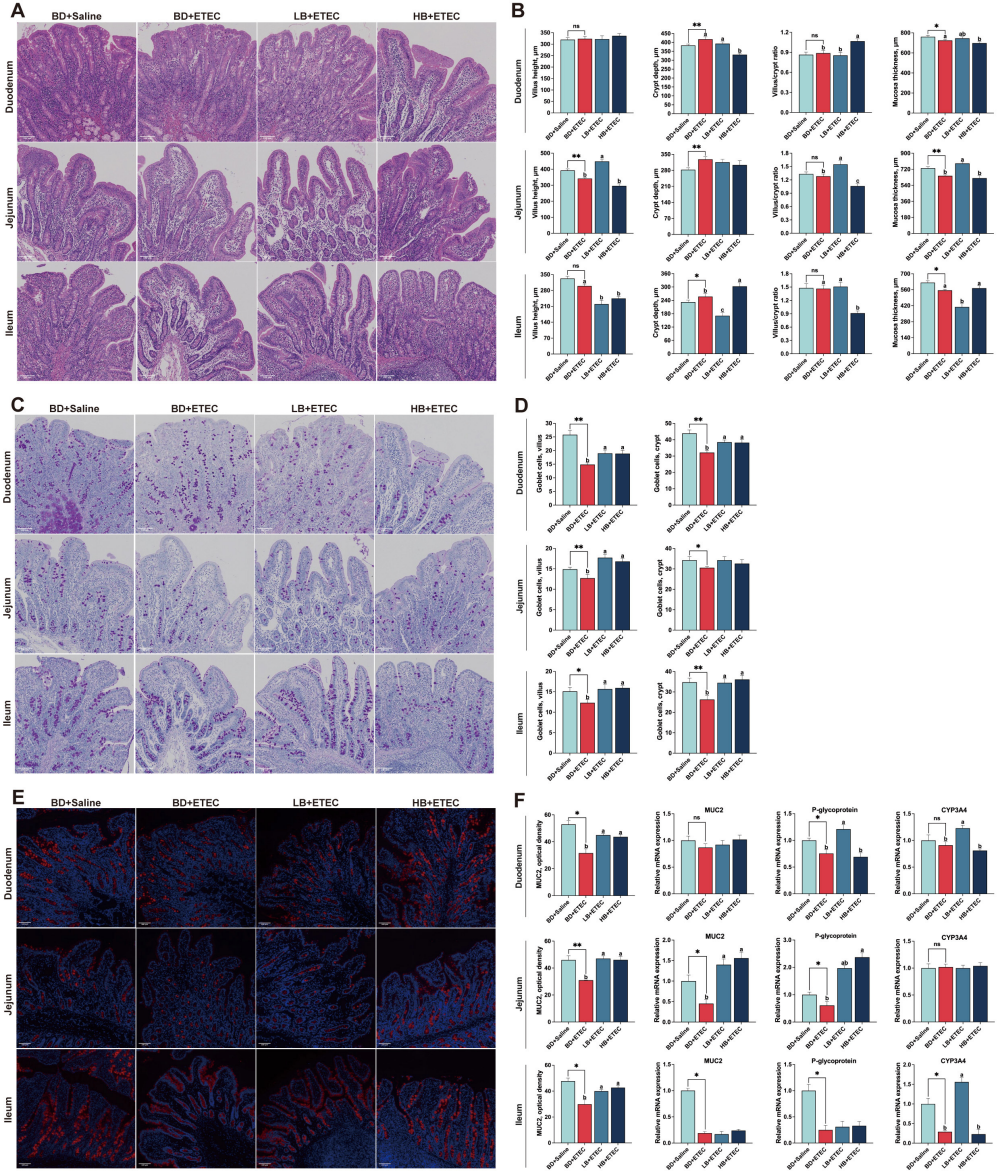
Effect of dietary berberine on the small intestinal histomorphology, goblet cells in villus and crypts, and intestinal chemical barrier of weaned piglets oral challenged with ETEC. **(A)** Representative HE staining of small intestinal tissue. **(B)** Villus height, crypt depth, the villus/crypt ratio, and mucosa thickness of the small intestine. **(C)** Representative goblet cells with AB-PAS staining in small intestinal tissue. **(D)** Goblet cell number in intestinal villus and crypts. **(E)** Representative immunofluorescence staining of mucin 2 (MUC2) in small intestinal tissue. **(F)** Optical density of MUC2, and the relative mRNA expressions of MUC2, P-glycoprotein, and CYP3A4. *BD + Saline vs BD + ETEC, *P < 0.05, **P < 0.01; ^a, b, c^BD + ETEC vs. LB + ETEC vs. HB + ETEC, different superscripts indicate significant differences among groups (*P* < 0.05); BD + Saline, basal diet with saline orally administered to piglets; BD + ETEC, basal diet with ETEC orally administered to piglets; LB + ETEC, basal diet with 0.05% berberine, ETEC orally administered to piglets; HB + ETEC, basal diet with 0.1% berberine, ETEC orally administered to piglets.

In the duodenum, the crypt depth in the BD + ETEC group was significantly higher than that in the BD + Saline group and the mucosal thickness was significantly lower than that in the BD + Saline group (*P* < 0.05). Crypt depth and mucosal thickness were significantly reduced in the HB + ETEC group and the villus/crypt ratio was significantly higher than that in the BD + ETEC group (*P* < 0.05). In the jejunum, compared with the BD + Saline group, jejunal villus height and mucosal thickness were higher and crypt depth was lower in the BD + ETEC group (*P* < 0.05). In contrast, villus height, villus/crypt ratio, and mucosal thickness were higher in the LB + ETEC group and the villus/crypt ratio was lower in the HB + ETEC group than in the BD + ETEC group (*P* < 0.05). In the ileum, crypt depth was higher and mucosal thickness was lower in the BD + ETEC than in the BD + Saline group (*P* < 0.05). The villus height, crypt depth, and mucosal thickness in the LB + ETEC and HB + ETEC groups were lower than those in the BD + ETEC group (*P* < 0.05) (Figures 2A, B).

### 3.3 Goblet cells in villus and crypts

We quantified the number of goblet cells in intestinal villi and crypts (Figures 2C, D). In the duodenum, compared to the BD + Saline group, the number of goblet cells in the villi and crypts in the BD + ETEC group was significantly lower (*P* < 0.05). Compared to the BD + ETEC group, the goblet cell number was increased in both the villi and crypts in the LB + ETEC and HB + ETEC groups (*P* < 0.05). In the jejunum, the number of goblet cells in the villi and crypts was significantly lower in the BD + ETEC group than in the BD + Saline group (*P* < 0.05). The number of goblet cells in the villi was significantly higher in the LB + ETEC and HB + ETEC groups than in the BD + ETEC group (*P* < 0.05). In the ileum, goblet cell numbers in both the villi and crypts were significantly lower in the BD + ETEC group than in the BD + Saline group (*P* < 0.05). Conversely, goblet cell numbers in both villi and crypts were significantly higher in the LB + ETEC and HB + ETEC groups than in the BD + ETEC group (*P* < 0.05) (Figure 2D).

### 3.4 Intestinal chemical barrier

To investigate differences in the chemical barrier of the small intestine, we evaluated the optical density of MUC2 and the expression of MUC2, P-glycoprotein, and CYP3A4 (Figures 2E, F). In the duodenum, the optical density of MUC2 and P-glycoprotein expression were lower in the BD + ETEC group than in the BD + Saline group (*P* < 0.05). Compared with the BD + ETEC group, the optical density of MUC2 in the LB + ETEC and HB + ETEC groups was significantly higher, along with the expression of P-glycoprotein and CYP3A4 in the LB + ETEC group (*P* < 0.05). In the jejunum, the optical density of MUC2 and the expression of MUC2 and P-glycoprotein were lower in the BD + ETEC than in the BD + Saline group (*P* < 0.05). Additionally, there was a noticeable increase in the optical density of MUC2 and MUC2 expression in the LB + ETEC and HB + ETEC groups, as well as a rise in P-glycoprotein expression in the HB + ETEC group compared to in the BD + ETEC group (*P* < 0.05). In the ileum, the optical density of MUC2 and expression P-glycoprotein and CYP3A4 were lower in the BD + ETEC group than in the BD + Saline group (*P* < 0.05). Moreover, compared to the BD + ETEC group, the LB + ETEC and HB + ETEC groups showed a marked increase in the optical density of MUC2, along with elevated CYP3A4 expression (*P* < 0.05) (Figure 2F).

### 3.5 Intestinal epithelial cell apoptotic status

**Figure 3:**
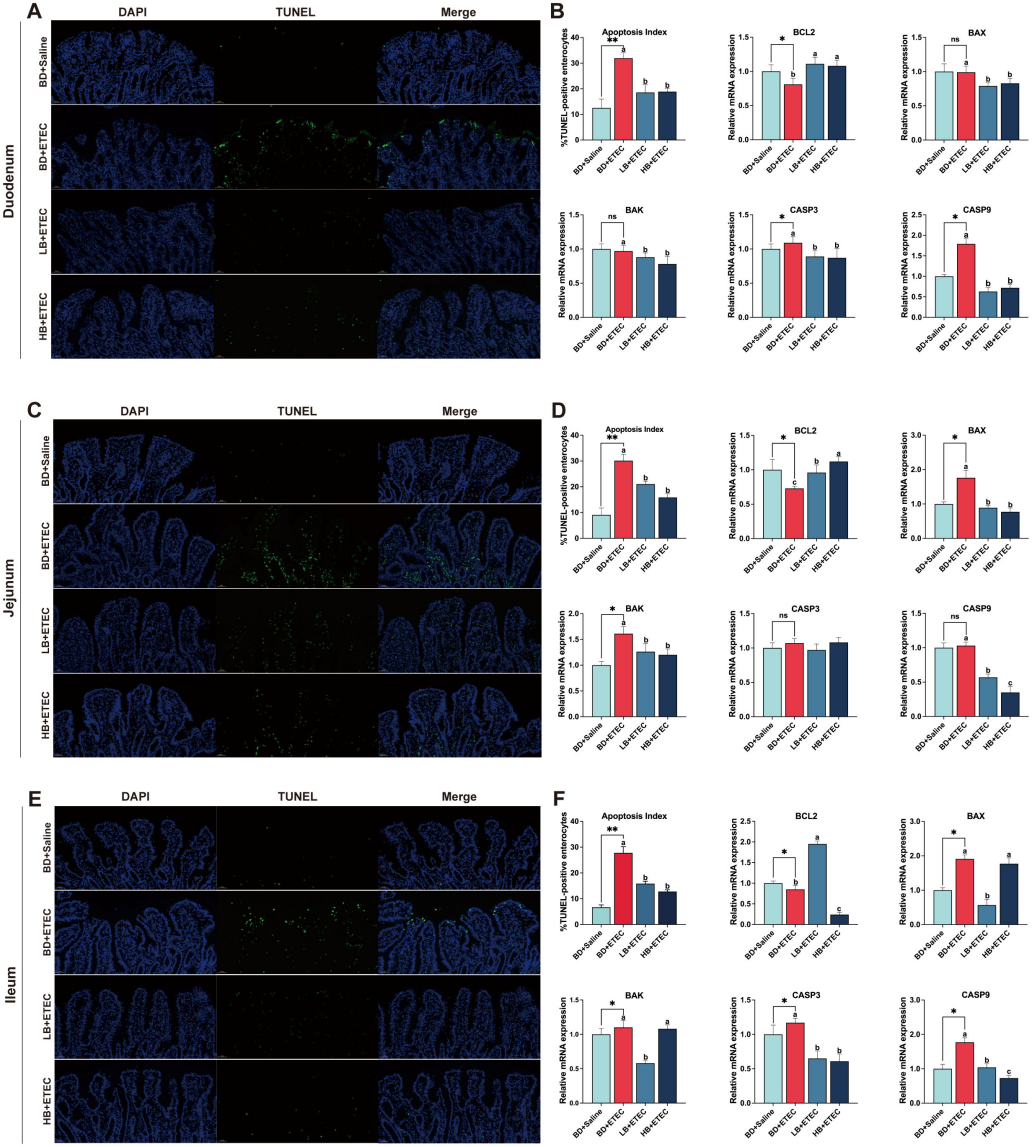
Effects of dietary berberine on the apoptosis status and apoptosis-related gene expression of intestinal epithelial cells in piglets orally challenged with ETEC. **(A)** Representative paraffin section of duodenum stained with TUNEL. **(B)** mRNA expressions of BCL2, BAX, BAK, CASP3, and CASP9 in the duodenum. **(C)** Representative paraffin section of jejunum stained with TUNEL. **(D)** mRNA expressions of BCL2, BAX, BAK, CASP3, and CASP9 in the jejunum. **(E)** Representative paraffin section of ileum stained with TUNEL. **(F)** mRNA expressions of BCL2, BAX, BAK, CASP3, and CASP9 in the ileum. *BD + Saline vs BD + ETEC, *P < 0.05, **P < 0.01; ^a, b, c^BD + ETEC vs. LB + ETEC vs. HB + ETEC, different superscripts indicate significant differences among groups (*P* < 0.05); BD + Saline, basal diet with saline orally administered to piglets; BD + ETEC, basal diet with ETEC orally administered to piglets; LB + ETEC, basal diet with 0.05% berberine, ETEC orally administered to piglets; HB + ETEC, basal diet with 0.1% berberine, ETEC orally administered to piglets.

TUNEL staining revealed the apoptotic state of the epithelial cells (Figure 3). In the duodenum, the expression of CASP3 and CASP9 was significantly increased, whereas that of BCL2 was decreased in the BD + ETEC compared to the BD + Saline group (*P* < 0.05). Compared to the BD + ETEC group, there was a significant decrease in the apoptosis index and expression of BAX, BAK, CASP3, and CASP9, whereas BCL2 was significantly increased in the LB + ETEC and HB + ETEC groups (*P* < 0.05) (Figures 3A, B). In the jejunum, the BD + ETEC group demonstrated a notable increase in the apoptosis index and expression of BAX and BAK, along with a decrease in BCL2, compared to the BD + Saline group (*P* < 0.05). The LB + ETEC and HB + ETEC groups displayed a decrease in the apoptosis index and expression levels of BAX, BAK, and CASP9, with an increase in BCL2 compared to the BD + ETEC group (*P* < 0.05) (Figures 3C, D). In the ileum, the apoptosis index and expression of BAX, BAK, CASP3, and CASP9 in the BD + ETEC group were significantly increased, whereas that of BCL2 was significantly decreased compared to that in the BD + Saline group (*P* < 0.05). Furthermore, compared with the BD + ETEC group, the apoptosis index and expression of CASP3 and CASP9 were significantly decreased in the LB + ETEC and HB + ETEC groups; BAX and BAK were significantly decreased in the LB + ETEC group and BCL2 was significantly increased in the LB + ETEC group (*P* < 0.05) (Figures 3E, F).

### 3.6 Intestinal physical barrier

**Figure 4:**
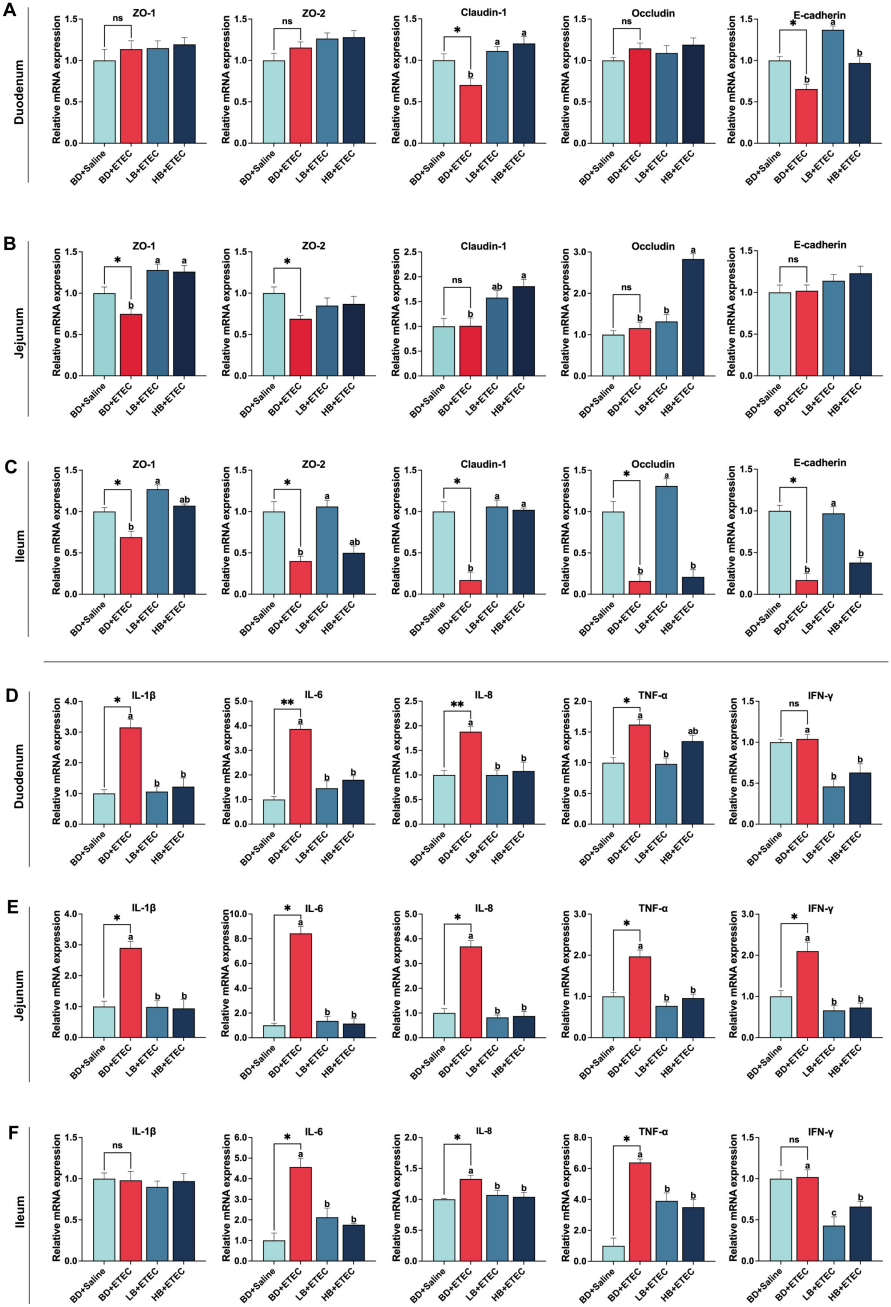
Effect of dietary berberine on the intestinal physical barrier and immune barrier of weaned piglets oral challenged with ETEC. The relative mRNA expressions of zonula occludins-1 (ZO-1), zonula occludins-2 (ZO-2), Claudin 1, Occludin, and E-cadherin in the duodenum **(A)**, jejunum **(B)**, and ileum **(C)**. The relative mRNA expressions of interleukin-1β (IL-1β), interleukin-6 (IL-6), interleukin-8 (IL-8), tumor necrosis factor-α (TNF-α), and Interferon-γ (IFN-γ) in the duodenum **(D)**, jejunum **(E)**, and ileum **(F)**. *BD + Saline vs BD + ETEC, *P < 0.05, **P < 0.01; ^a, b, c^BD + ETEC vs. LB + ETEC vs. HB + ETEC, different superscripts indicate significant differences among groups (*P* < 0.05); BD + Saline, basal diet with saline orally administered to piglets; BD + ETEC, basal diet with ETEC orally administered to piglets; LB + ETEC, basal diet with 0.05% berberine, ETEC orally administered to piglets; HB + ETEC, basal diet with 0.1% berberine, ETEC orally administered to piglets.

The mRNA expression levels of physical barrier-relevant genes in the small intestine are shown in Figures 4A–C. In the duodenum, the expressions of Claudin 1 and E-cadherin were notably decreased in the BD + ETEC than BD + Saline group (*P* < 0.05). Compared to the BD + ETEC group, claudin 1 expression was notably increased in the LB + ETEC and HB + ETEC groups, and E-cadherin expression was increased in the LB + ETEC group (*P* < 0.05) (Figure 4A). In the jejunum, the expressions of ZO-1 and ZO-2 were notably decreased in the BD + ETEC than BD + Saline group (*P* < 0.05). Compared to the BD + ETEC group, ZO-1 expression in the LB + ETEC and HB + ETEC groups and the expressions of Claudin 1 and Occludin in the HB + ETEC group were notably increased (*P* < 0.05) (Figure 4B). In the ileum, the expression of ZO-1, ZO-2, Claudin 1, Occludin, and E-cadherin was notably lower in the BD + ETEC group than in the BD + Saline group (*P* < 0.05). Compared to the BD + ETEC group, the expression of ZO-1, ZO-2, Occludin, and E-cadherin was notably increased in the LB + ETEC group, and claudin 1 was notably increased in the LB + ETEC and HB + ETEC groups (*P* < 0.05) (Figure 4C).

### 3.7 Intestinal immune barrier

In the duodenum, the expressions of IL-1β, IL-6, IL-8, and TNF-α were notably increased in the BD + ETEC than in the BD + Saline group (*P* < 0.05). Compared to the BD + ETEC group, the IL-1β, IL-6, IL-8, and IFN-γ expressions in the LB + ETEC and HB + ETEC groups and TNF-α expression in the LB + ETEC group were notably decreased (*P* < 0.05) (Figure 4D). In the jejunum, compared to the BD + Saline group, the expressions of IL-1β, IL-6, IL-8, TNF-α, and IFN-γ were notably increased in the BD + ETEC group (*P* < 0.05). The expressions of IL-1β, IL-6, IL-8, TNF-α, and IFN-γ were significantly decreased in the LB + ETEC and HB + ETEC groups compared to in the BD + ETEC group (*P* < 0.05) (Figure 4E). In the ileum, the expressions of IL-6, IL-8, and TNF-α were notably increased in the BD + ETEC group compared to in the BD + Saline group (*P* < 0.05). Compared with the BD + ETEC group, the expressions of IL-6, IL-8, TNF-α, and IFN-γ were notably decreased in the LB + ETEC and HB + ETEC groups (*P* < 0.05) (Figure 4F).

### 3.8 VFA concentrations in colonic content

The VFA concentrations in the colonic digesta are summarized in Table 2. Total VFA, acetic acid, propionic acid, butyric acid, valeric acid, and isovaleric acid levels were notably lower in the BD + ETEC group than in the BD + Saline group (*P* < 0.05). Conversely, total VFA, acetic acid, propionic acid, valeric acid, and isovaleric acid levels were notably higher in the LB + ETEC group compared to the BD + ETEC group (*P* < 0.05). Compared to the BD + Saline group, total VFA, acetic acid, butyric acid, and valeric acid were significantly decreased in the HB + ETEC group (*P* < 0.05). The concentrations of total VFA, acetic acid, propionic acid, and valeric acid levels were not significantly different between the HB + ETEC and BD + Saline group (*P* < 0.05) (Table 2).

**Table 2:** Effect of berberine on the VFA concentrations in colonic content of weaned piglets orally challenged with ETEC.

**Measure**	**Experimental diet**				**SEM**	***P*-value**
	**BD** + **Saline**	**BD** + **ETEC**	**LB** + **ETEC**	**HB** + **ETEC**		
Total VFA, μmoL/g	84.77^a^	63.13^b^	82.90^a^	67.55^b^	2.64	< 0.05
Acetic acid, μmoL/g	46.26^a^	34.31^b^	46.52^a^	33.84^b^	1.43	< 0.05
Propionic acid, μmoL/g	30.21^a^	16.71^b^	29.22^a^	25.25^ab^	1.68	< 0.05
Butyric acid, μmoL/g	10.56^a^	6.47^b^	5.83^b^	6.11^b^	0.57	< 0.05
Valeric acid, μmoL/g	0.87^a^	0.43^b^	1.21^a^	0.76^b^	0.09	< 0.05
Isovaleric acid, μmoL/g	1.09^b^	0.52^c^	1.41^a^	1.16^b^	0.11	< 0.05

^a, b, c^Values with different superscripts in the row indicate significant differences (*P* < 0.05).

### 3.9 Intestinal biological barrier

**Figure 5:**
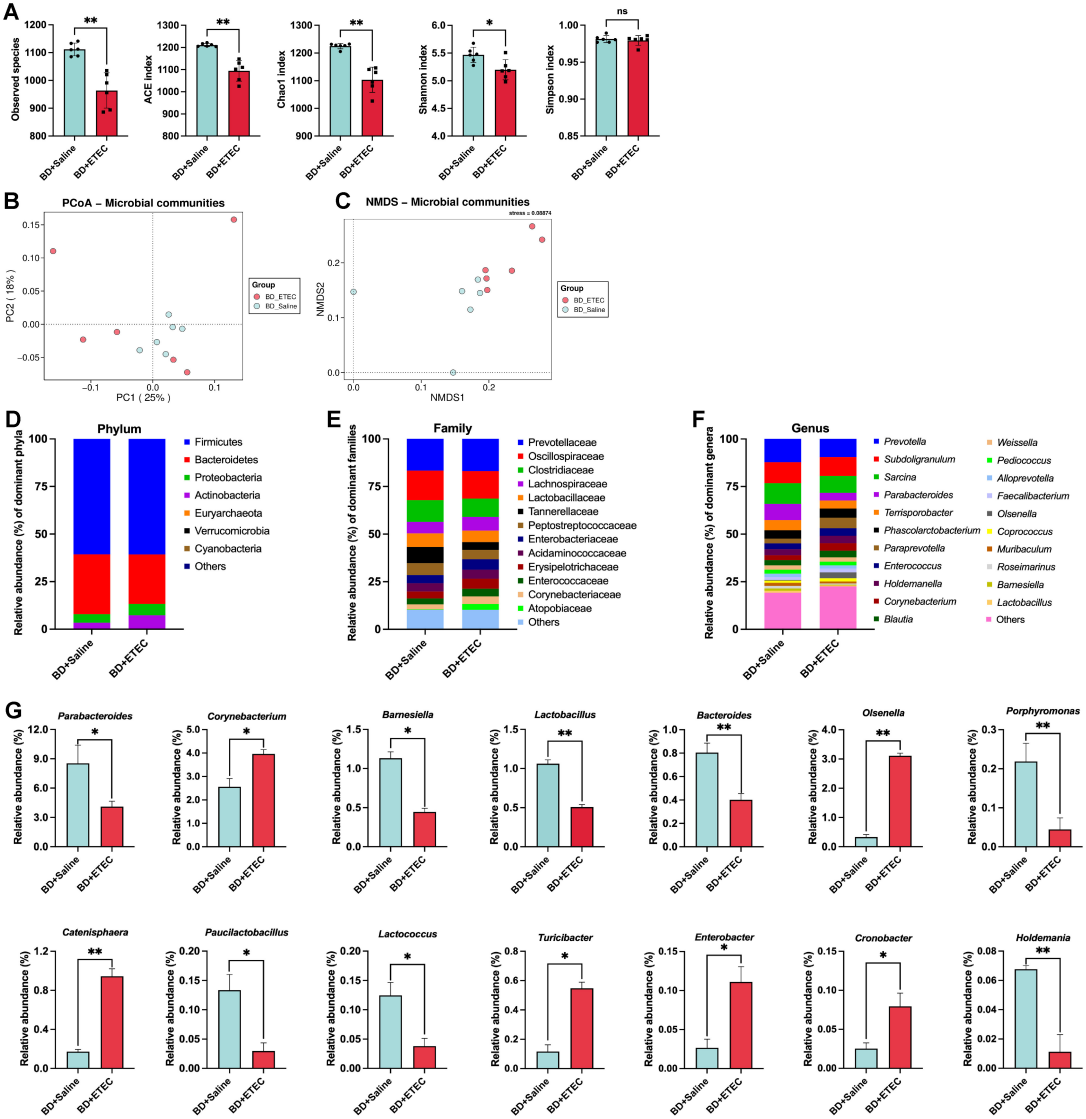
Effects of ETEC infection on the intestinal microbiota of weaned piglets and its correlation with small intestinal barrier function marker genes. **(A)** Richness (ACE and Chao1) and diversity (Shannon and Simpson) of the intestinal microbiota of weaned piglets. **(B)** PCoA analysis. **(C)** NMDS analysis. The composition and structure of the intestinal microbiota in weaned piglets (the relative abundance > 1%) at the **(D)** phylum level, **(E)** family level, **(F)** and genus levels. **(G)** Significant differences in the relative abundances of genera between two groups. *BD + Saline vs. BD + ETEC, *P < 0.05, **P < 0.01; BD + Saline, basal diet with saline orally administered to piglets; BD + ETEC, basal diet with ETEC orally administered to piglets.

To evaluate the impact of ETEC infection on the intestinal biological barrier in piglets, we analyzed the composition and structure of the intestinal microbiota in the BD + Saline and BD + ETEC groups (Figure 5). Alpha and beta diversities were used to evaluate the richness and diversity of the intestinal microbiota. In comparison with the BD + Saline group, the observed species and ACE and Chao1 richness estimators were significantly lower in the BD + ETEC group. Additionally, Shannon diversity estimators significantly decreased in the BD + ETEC group (*P* < 0.05). The PCoA and NMDS analyses based on Bray-Curtis distances revealed significant differences in the microbial community structure between the BD + Saline and BD + ETEC groups (Figures 5B, C). At the phylum level, Firmicutes and Bacteroidetes were the predominant phyla in the piglet intestinal microbiota, followed by Proteobacteria and Actinobacteria (Figure 5D). At the family level, 13 families with a relative abundance of >1% were dominant (Figure 5E). At the genus level, 21 genera with a relative abundance of >1% were dominant (Figure 5F). The abundances of *Parabacteroides, Barnesiella, Lactobacillus, Bacteroides, Porphyromonas, Paucilactobacillus, Lactococcus*, and *Holdemania* were significantly lower and the abundances of *Corynebacterium, Olsenella, Catenisphaera, Turicibacter, Enterobacter*, and *Cronobacter* were significantly higher in the BD + ETEC group than in the BD + Saline group (*P* < 0.05) (Figure 5G).

**Figure 6:**
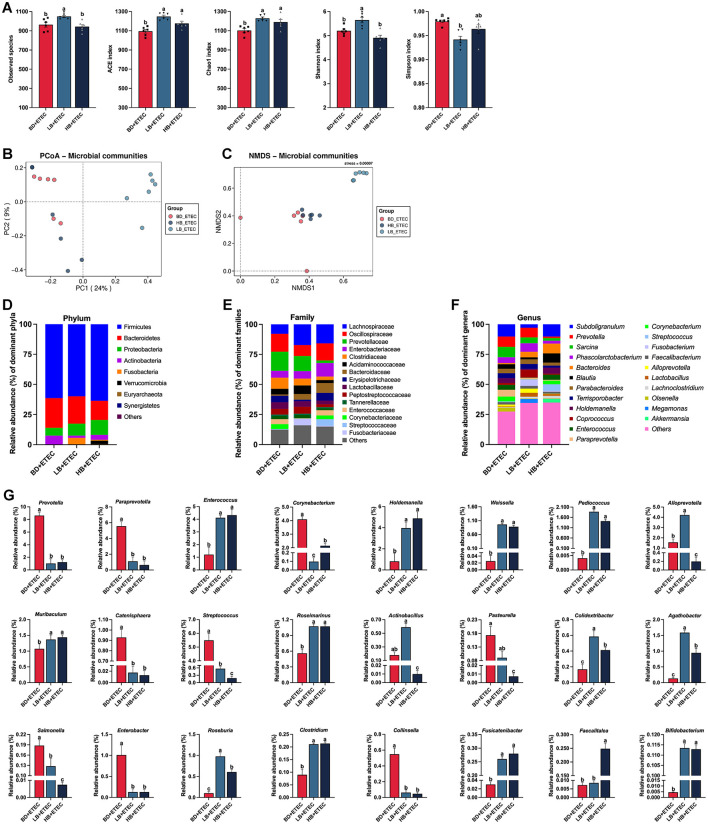
Effects of dietary berberine on the intestinal microbiota of weaned piglets oral challenged with ETEC and its correlation with small intestinal barrier function marker genes. **(A)** Richness (ACE and Chao1) and diversity (Shannon and Simpson) of the intestinal microbiota of weaned piglets. **(B)** PCoA analysis. **(C)** NMDS analysis. The composition and structure of the intestinal microbiota in weaned piglets (the relative abundance of > 1%) at **(D)** phylum, **(E)** family, and **(F)** genus levels. **(G)** Significant differences in the relative abundances of genera among three groups. ^a, b, c^BD + ETEC vs. LB + ETEC vs. HB + ETEC, different superscripts indicate significant differences among groups (*P* < 0.05); BD + ETEC, basal diet with ETEC orally administered to piglets; LB + ETEC, basal diet with 0.05% berberine, ETEC orally administered to piglets; HB + ETEC, basal diet with 0.1% berberine, ETEC orally administered to piglets.

The experimental results on the composition and structure of the intestinal microbiota in the BD + ETEC, LB + ETEC, and HB + ETEC groups are shown in Figure 6. As shown in Figure 6A, compared to the BD + ETEC group, the ACE and Chao1 indices were significantly higher in the LB + ETEC and HB + ETEC groups. Additionally, the observed species and Shannon index were significantly higher and the Simpson index was significantly lower in the LB + ETEC group (*P* < 0.05). The PCoA and NMDS analyses based on Bray-Curtis distances revealed significant differences in the microbial community structure between the BD + ETEC and LB + ETEC groups (see Figures 6B, C). At the phylum level, Firmicutes, Bacteroidetes, and Proteobacteria were dominant in the piglet intestinal microbiota, followed by Actinobacteria and Fusobacteria (Figure 6D). At the family level, 15 families with a relative abundance of >1% were dominant (Figure 6E). At the genus level, 22 genera with a relative abundance of >1% were dominant (Figure 6F). The relative abundances of *Prevotella, Paraprevotella, Corynebacterium, Catenisphaera, Streptococcus, Salmonella, Enterobacter*, and *Collinsella* were significantly lower and the abundances of *Enterococcus, Holdemanella, Weissella, Pediococcus, Muribaculum, Roseimarinus, Colidextribacter, Agathobacter, Roseburia, Clostridium, Fusicatenibacter*, and *Bifidobacterium* were significantly higher in the BD + ETEC group than LB + ETEC and HB + ETEC group (*P* < 0.05) (Figure 6G).

### 3.10 Correlations between intestinal microbiota and intestinal barrier function

**Figure 7:**
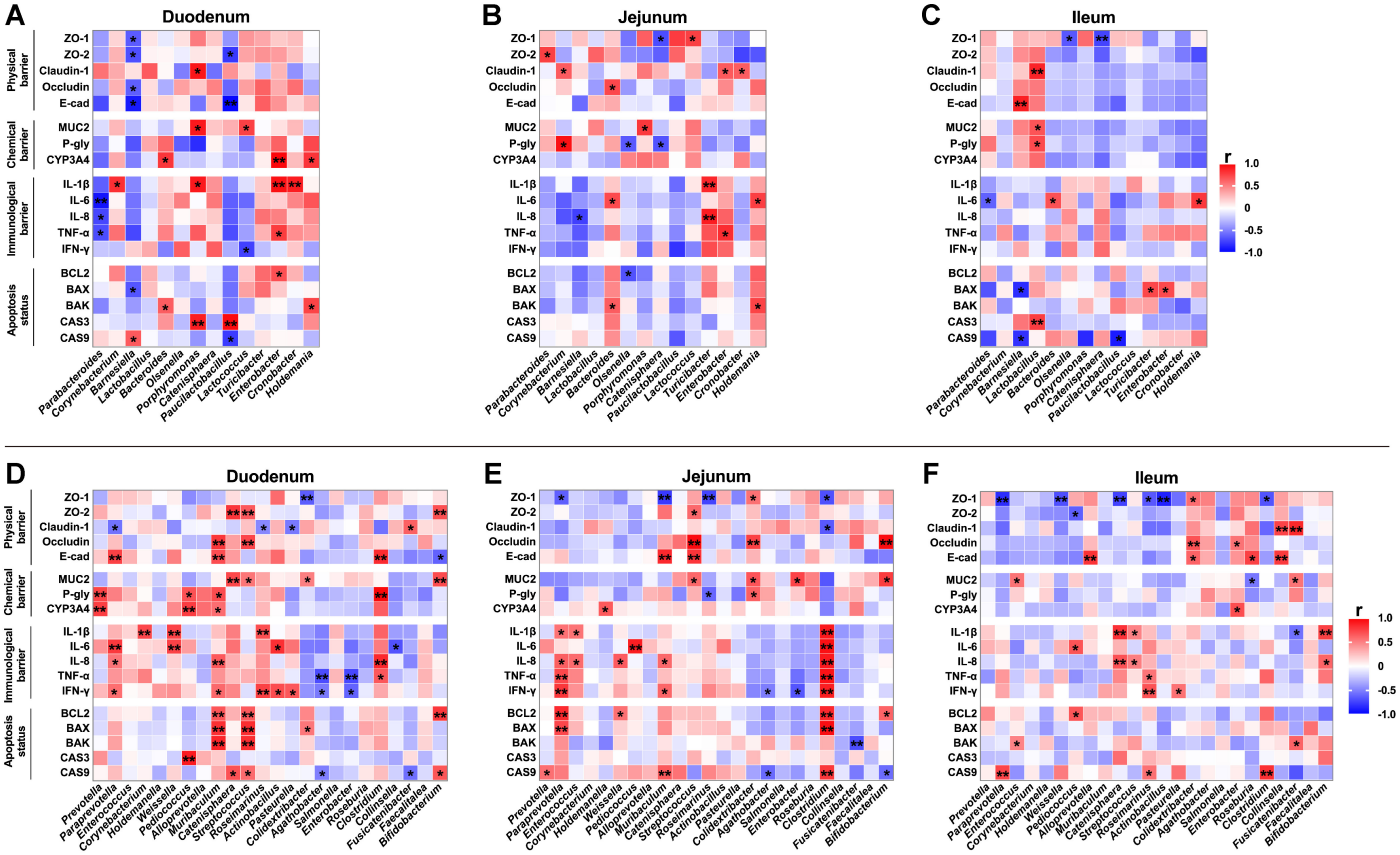
Correlation analysis of intestinal microbiota (genus level) and the relative mRNA expressions of marker genes for intestinal barrier function. The correlation between bacterial genera showing significant changes and the relative mRNA expressions of intestinal barrier function marker genes in the **(A)** duodenum, **(B)** jejunum, and **(C)** ileum between the BD + Saline and BD + ETEC groups. The correlation between changes in intestinal microbiota and the relative mRNA expressions of intestinal barrier function marker genes in the **(D)** duodenum, **(E)** jejunum, and **(F)** ileum among the BD + ETEC, LB + ETEC, and HB + ETEC groups. BD + Saline, basal diet with saline orally administered to piglets; BD + ETEC, basal diet with ETEC orally administered to piglets; LB + ETEC, basal diet with 0.05% berberine, ETEC orally administered to piglets; HB + ETEC, basal diet with 0.1% berberine, ETEC orally administered to piglets; Red indicates a positive correlation and blue indicates negative correlation. *Significant correlation *P* < 0.05, **significant correlation *P* < 0.0l.

To investigate the correlation between alterations in the microbial community structure and changes in intestinal barrier function induced by ETEC infection, we analyzed the relationship between bacterial genera showing significant changes and gut barrier function marker genes in the BD + Saline and BD + ETEC groups (Figures 7A–C). In the duodenum, the abundances of *Barnesiella* and *Paucilactobacillus* were significantly negatively correlated with intestinal physical barriers (ZO-1, ZO-2, Occludin, E-cadherin). Conversely, the relative abundances of *Bacteroides, Porphyromonas, Lactococcus, Enterobacter*, and *Holdemania* showed a significant positive correlation with intestinal chemical barriers (MUC2 and CYP3A4). Furthermore, the abundance of *Corynebacterium, Porphyromonas, Enterobacter*, and *Cronobacter* exhibited a significant positive relationship, while the abundance of *Parabacteroides* and *Lactococcus* showed a significant negative correlation with intestinal immunological barriers (IL-1β, IL-6, IL-8, TNF-α, and IFN-γ). Lastly, the abundances of *Barnesiella, Bacteroides, Porphyromonas, Paucilactobacillus, Enterobacter*, and *Holdemania* demonstrated significant positive correlations with the apoptosis status (BCL2, BAX, BAK, CASP3, and CASP9), whereas the abundances of *Barnesiella* and *Paucilactobacillus* were significantly negatively correlated (Figure 7A). In the jejunum, the relative abundances of *Parabacteroides, Corynebacterium, Bacteroides, Lactococcus, Enterobacter*, and *Cronobacter* were significantly positively correlated with the intestinal physical barriers (ZO-1, ZO-2, Claudin-1, and Occludin). Additionally, the abundances of *Corynebacterium* and *Porphyromonas* exhibited significant positive correlations with intestinal chemical barriers (MUC2 and P-glycoprotein), whereas the abundances of *Olsenella* and *Catenisphaera* were significantly negatively correlated. Furthermore, the relative abundances of *Bacteroides, Turicibacter, Enterobacter*, and *Holdemania* displayed significant positive correlations with intestinal immunological barriers (IL-1β, IL-6, IL-8, and TNF-α), while the abundance of *Barnesiella* showed a significant negative correlation. Lastly, the abundances of *Bacteroides* and *Holdemania* demonstrated significant positive correlations with the apoptosis status (BCL2 and BAK), whereas the abundance of *Olsenella* was significantly negatively correlated (Figure 7B). In the ileum, the relative abundances of *Barnesiella* and *Lactobacillus* were significantly positively correlated with the intestinal physical barriers (ZO-1, Claudin-1, and E-cadherin), whereas the abundances of *Olsenella* and *Catenisphaera* were significantly negatively correlated. Additionally, the abundance of *Lactobacillus* was significantly positively correlated with intestinal chemical barriers (MUC2 and P-glycoprotein). Moreover, the abundances of *Bacteroides* and *Holdemania* were significantly positively correlated with intestinal immunological barriers (IL-6), while the abundance of *Parabacteroides* was significantly negatively correlated. Lastly, the abundances of *Lactobacillus, Turicibacter*, and *Enterobacter* were significantly positively correlated with apoptosis status (BAX, CASP3, and CASP9), while the abundances of *Barnesiella* and *Paucilactobacillus* were significantly negatively correlated (Figure 7C).

To investigate the mechanism of berberine in alleviating intestinal barrier damage caused by ETEC infection, we analyzed the correlation between changes in intestinal microbiota and gut barrier function in BD + ETEC, LB + ETEC, and HB + ETEC groups (Figures 7D–F). In the duodenum, the relative abundances of *Paraprevotella, Muribaculum, Catenisphaera, Streptococcus, Clostridium, Fusicatenibacter*, and *Bifidobacterium* were significantly and positively correlated with intestinal physical barriers (ZO-1, ZO-2, Claudin-1, Occludin, and E-cadherin). Conversely, the abundances of *Paraprevotella, Roseimarinus, Pasteurella, Colidextribacter*, and *Bifidobacterium* were significantly negatively correlated. Additionally, the abundances of *Prevotella, Pediococcus, Muribaculum, Catenisphaera, Streptococcus, Colidextribacter, Clostridium*, and *Bifidobacterium* were significantly positively correlated with intestinal chemical barriers (MUC2, P-glycoprotein, and CYP3A4). Furthermore, the abundance of *Paraprevotella, Corynebacterium, Weissella, Muribaculum, Roseimarinus, Actinobacillus, Pasteurella*, and *Clostridium* were significantly positively correlated with intestinal immunological barriers (IL-1β, IL-6, IL-8, TNF-α, and IFN-γ), while the abundance of *Agathobacter, Enterobacter*, and *Collinsella* were significantly negatively correlated. Lastly, the abundances of *Paraprevotella, Muribaculum, Catenisphaera, Streptococcus, Colidextribacter*, and *Bifidobacterium* were significantly positively correlated with apoptosis status (BCL2, BAX, BAK, CASP3, and CASP9), while the abundances of *Agathobacter*, and *Fusicatenibacter* were significantly negatively correlated with CASP9 (Figure 7D). In the jejunum, the relative abundances of *Muribaculum, Streptococcus, Colidextribacter, Enterobacter*, and *Bifidobacterium* showed significant positive correlations, whereas the abundances of *Paraprevotella, Muribaculum, Roseimarinus*, and *Clostridium* were significantly negatively correlated with intestinal physical barriers (ZO-1, ZO-2, Claudin-1, Occludin, and E-cadherin). Similarly, the abundances of *Holdemanella, Streptococcus, Colidextribacter, Enterobacter*, and *Bifidobacterium* were significantly positively correlated, whereas the abundance of *Roseimarinus* was significantly negatively correlated with intestinal chemical barriers (MUC2, P-glycoprotein, and CYP3A4). Moreover, the abundance of *Paraprevotella, Enterococcus, Weissella, Pediococcus, Muribaculum*, and *Clostridium* displayed significant positive correlations, while the abundance of *Agathobacter* and *Enterobacter* showed significant negative correlations with intestinal immunological barriers (IL-1β, IL-6, IL-8, TNF-α, and IFN-γ). Lastly, the abundances of *Prevotella, Paraprevotella, Weissella, Muribaculum, Clostridium*, and *Bifidobacterium* demonstrated significant positive correlations, while the abundances of *Agathobacter, Fusicatenibacter*, and *Bifidobacterium* were significantly negatively correlated with the apoptosis status (BCL2, BAX, BAK, and CASP9) (Figure 7E). In the ileum, the relative abundances of *Alloprevotella, Colidextribacter, Enterobacter, Roseburia, Collinsella*, and *Fusicatenibacter* were significantly positively correlated with the intestinal physical barriers (ZO-1, ZO-2, Claudin-1, Occludin, and E-cadherin), whereas the abundances of *Paraprevotella, Weissella, Pediococcus, Catenisphaera, Roseimarinus, Actinobacillus*, and *Clostridium* were significantly negatively correlated. Additionally, the abundance of *Enterococcus, Enterobacter*, and *Fusicatenibacter* was significantly positively correlated with intestinal chemical barriers (MUC2, P-glycoprotein, and CYP3A4), whereas the abundance of *Roseburia* exhibited a significant negative correlation. Furthermore, *Pediococcus, Catenisphaera, Streptococcus, Roseimarinus, Pasteurella*, and *Bifidobacterium* were significantly positively correlated with intestinal immunological barriers (IL-1β, IL-6, IL-8, TNF-α, and IFN-γ), while *Fusicatenibacter* showed a significant negative correlation. Lastly, the abundances of *Paraprevotella, Enterococcus, Pediococcus, Roseimarinus, Clostridium*, and *Fusicatenibacter* were significantly positively correlated with apoptosis status (BCL2, BAK, and CASP9) (Figure 7F).

## 4 Discussion

ETEC is often present in the intestinal microbiota and is a prevalent pathogen. It causes diarrhea in piglets and children, particularly during weaning and neonatal development (27). The release of adhesion and enterotoxin by ETEC in the intestine disrupts the intestinal flora, damages the intestinal epithelial barrier, increases intestinal permeability, and triggers inflammatory stress, ultimately leading to diarrhea (27–29). Our prior research results indicated that berberine has the potential to enhance the makeup and arrangement of intestinal microbiota in piglets after weaning. Furthermore, it exhibits noteworthy antibacterial activity against *E. coli* in laboratory settings (22, 25). The current study aimed to investigate the mechanism by which berberine alleviates damage to intestinal barrier function in a weaned piglet model infected with ETEC.

Previous studies have shown that ETEC leads to significant damage to the small intestinal mucosal barrier function (29, 30), resulting in increased epithelial permeability (31). Circulating markers, such as plasma DAO, d-lactate, and endotoxin, which assess the extent of damage and repair of intestinal mucosal barrier integrity (30, 32, 33). In the current study, plasma DAO, D-lactate, and endotoxin levels were elevated in piglets from the BD + ETEC group. Conversely, the levels of DAO and D-lactate were decreased in the LB + ETEC and HB + ETEC groups, and endotoxin was decreased in the LB + ETEC group. These findings suggest that ETEC infection increases intestinal permeability in piglets (30, 31, 33). Moreover, our results indicate that berberine can mitigate the increased intestinal permeability caused by ETEC infection in piglets.

The primary site of digestion and absorption is the small intestine (34). Important variables that indicate the digestive and absorptive efficiency of the small intestine consist of the villus height, crypt depth, and villus-to-crypt ratio (35–37), with a healthy gut in pigs is characterized by a higher villus height, villus/crypt ratio, and shallow crypts (34, 37, 38). In this study, ETEC challenge (BD + ETEC) decreased villus height in the jejunum and increased crypt depth in the duodenum, jejunum, and ileum. However, it did not significantly affect the villus/crypt ratio. These results are consistent with those of recent studies on pigs (34–36). The application of berberine (LB + ETEC) increased villus height and the villus/crypt ratio in the jejunum, suggesting that berberine can alleviate ETEC-induced jejunal tissue lesions. In piglets, ETEC infection can damage the mucosal layer of the intestinal mucosa (37, 39). We observed a significant decrease in mucosal thickness in the duodenum, jejunum, and ileum following ETEC challenge (BD + ETEC). However, the application of berberine (LB + ETEC) increased mucosal thickness, specifically in the jejunum (Figure 2B). These findings align with those of previous studies (29, 37, 39, 40), suggesting that berberine might protect against morphological damage to the intestines caused by ETEC infection, with a particular impact on the jejunum.

In the intestine, goblet cells play a vital role in eliminating undigested food, microbes, and by-products produced by microbes through the secretion of mucins to form mucus layers. These layers are essential for maintaining the integrity of the intestinal epithelium (41). Goblet cells that produce mucins are integral to the non-specific intestinal barrier and offer partial protection against bacterial and fungal intrusions (39, 42). In this study, ETEC infection (BD + ETEC) decreased the number of goblet cells in the villi and crypts of the duodenum, jejunum, and ileum. Conversely, berberine treatment significantly increased the number of goblet cells in the villi and crypts of the duodenum, jejunum, and ileum (Figure 2D). These results are consistent with those of previous studies (39, 41–43). A decrease in the number of goblet cells may reduce mucin secretion in the mucosa, potentially damaging the mucosal barrier in piglets (42). This study suggests that a decrease in goblet cells in the small intestine of weaned piglets due to ETEC infection may have contributed to the observed reduction in mucosal thickness, leading to compromised intestinal mucosal barrier function. Berberine effectively alleviated the reduction in the number of goblet cells caused by ETEC infection in weaned piglets.

Building on our observation that ETEC infection leads to a decrease in goblet cells in the small intestine, we examined MUC2 expression in the same region as in piglets. MUC2 is the main gel-forming mucin secreted by goblet cells within the small intestine and is essential for preserving the integrity of the intestinal epithelial barrier (44, 45). ETEC infection (BD + ETEC) significantly decreased the optical density of MUC2 in the duodenum, jejunum, and ileum and reduced MUC2 expression in the jejunum and ileum. Conversely, dietary berberine significantly increased the optical density of MUC2 in the duodenum, jejunum, and ileum (Figure 2F). In the small intestine, MUC2 assembles into an intricate network of glycoprotein multimers that bacterial pathogens must traverse to successfully interact with epithelial cells (46–48). These findings suggest that a reduction in goblet cells leads to a decrease in MUC2 levels, which facilitates ETEC penetration through chemical barriers. Berberine appears to prevent ETEC invasion of intestinal epithelial cells by increasing MUC2 levels. P-gp and CYP3A4 serve as chemical barriers in the small intestine (49). P-glycoprotein is a membrane-bound transporter protein that protects against harmful substances. CYP3A4 enzymes are present in the small intestine and act as barriers against xenobiotics (49). In this study, ETEC infection (BD + ETEC) decreased P-glycoprotein expression in the duodenum, jejunum, and ileum and decreased CYP3A4 expression in the ileum. Conversely, dietary berberine significantly increased the expression of P-gp and CYP3A4 in the duodenum, jejunum, and ileum (Figure 2F). These findings are consistent with those of previous studies indicating that reduced expression of P-gp and CYP3A4 in the small intestine of piglets may contribute to the compromised intestinal mucosal barrier during ETEC infection. Berberine can mitigate this barrier damage (37, 50).

Enteric pathogens commonly induce programmed cell death, also known as apoptosis, to facilitate survival and spread during infection (43). When ETEC infection breaches the chemical defenses of the small intestine and comes into contact with intestinal epithelial cells, enterotoxins quickly trigger apoptosis in intestinal epithelial cells, leading to enhanced ETEC adherence (35, 43, 44, 51). In our study, ETEC infection (BD + ETEC) induced apoptosis in the duodenum, jejunum, and ileum of weaned piglets. However, berberine supplementation reduced intestinal epithelial cell apoptosis in the piglets (Figure 3). BCL2 family members are vital for the regulation of apoptotic processes. This group includes anti-apoptotic BCL2 proteins as well as the pro-apoptotic proteins BAX and BAK (52). During intrinsic apoptosis, the balance within the BCL2 family is disrupted by DNA damage, leading to caspase-9 activation. Caspase-9 initiates an apoptotic cascade that culminates in the cleavage of caspase-3 (CASP3) (53). Caspase-3 is a key effector caspase in apoptosis that is responsible for cleaving downstream caspases and driving the biochemical changes associated with cell death (53). In this study, ETEC challenge (BD + ETEC) decreased BCL2 expression in the duodenum, jejunum, and ileum, while increasing the expression of CASP3 and CASP9 in the duodenum. Additionally, it led to increased expression of BAX and BAK in the jejunum and elevated expression of BAX, BAK, CASP3, and CASP9 in the ileum. Berberine administration (LB + ETEC and HB + ETEC) increased BCL2 expression in the duodenum, jejunum, and ileum; decreased the expression of BAX, BAK, CASP3, and CASP9 in the duodenum; decreased the expression of BAX, BAK, and CASP9 in the jejunum; and decreased the expression of CASP3 and CASP9 in the ileum. These findings suggest that ETEC infection diminishes the anti-apoptotic capacity of weaned piglets, leading to the apoptosis of small intestinal epithelial cells. Berberine shows promise for mitigating apoptosis by enhancing its anti-apoptotic ability. These results were consistent with those of the TUNEL staining.

Our study demonstrates that ETEC infection leads to elevated intestinal permeability, histomorphological damage to the small intestine, decreased goblet cell count, compromised chemical barrier function, and induced apoptosis in intestinal epithelial cells. These findings motivated us to delve deeper into the effects of ETEC infection on the physical barrier function of the small intestine of piglets. Epithelial cells in the intestine form a tight organizational structure via a range of cell junctions, including tight junctions, adhesion junctions, and desmosomes. These components play a crucial role in providing resistance to pathogens, viruses, and toxins (28). These junctions work together to seal paracellular pathways (30). In the context of ETEC invasion, tight junction proteins (such as ZO-1, ZO-2, Claudin-1, and Occludin) and adhesion junction proteins (such as E-cadherin) are key to determining mucosal permeability. When these proteins break down, they allow substances, such as bacteria, toxins, and antigens, to pass from the lumen into the body, disrupting barrier function and leading to diarrhea (6, 29, 50). In this study, ETEC infection (BD + ETEC) decreased the expression of Claudin-1 and E-cadherin in the duodenum and decreased the expression of ZO-1 and ZO-2 in the jejunum. Furthermore, it decreased the expression of ZO-1, ZO-2, Claudin-1, Occludin, and E-cadherin in the ileum. The application of berberine (LB + ETEC) significantly increased the expression of these marker genes in the ileum (Figures 4A–C). Our findings align with those of a previous study that reported that ETEC can harm intestinal tight junctions and adhesion junctions (28). Reduced expression of ZO-1, ZO-2, Claudin-1, Occludin, and E-cadherin is associated with intestinal barrier dysfunction and increased intestinal permeability (28, 29, 54). Our results indicated that Berberine alleviates the damage to tight junctions and adhesion junctions in the ileum of weaned piglets caused by ETEC infection, which is consistent with the findings related to intestinal permeability.

A disrupted barrier facilitates the unrestricted and easy entry of ETEC into the mucosa, which can impair the normal functions of the body and the immune system. This often results in inflammatory responses (55). Cytokines play essential roles in controlling the integrity of the intestinal immune barrier. Indicators of the inflammatory reaction in the gut consist of IL-1β, IL-6, IL-8, TNF-α, and IFN-γ. Growing evidence supports the ability of ETECs to compromise the intestinal barrier in piglets by elevating the levels of pro-inflammatory cytokines (40, 56, 57). Consistent with these findings, in this study, ETEC infection (BD + ETEC) led to increased expressions of IL-1β, IL-6, IL-8, and TNF-α in the duodenum. Additionally, there was an increase in the expressions of IL-1β, IL-6, IL-8, TNF-α, and IFN-γ in the jejunum, while the expressions of IL-6, IL-8, and TNF-α were elevated in the ileum. Following berberine administration, the expression of these marker genes was notably reduced in the duodenum, jejunum, and ileum (Figures 4D–F). This indicates that BBR mitigates the damage to the small intestinal immune barrier in weaned piglets caused by ETEC infection.

VFAs are significant by-products of intestinal microbial metabolism and contribute to the maintenance of intestinal function (58). Acetic acid, propionic acid, and butyric acid play key roles in regulating inflammatory responses and maintaining intestinal barrier functions (37). Acetic and propionic acids function as energy sources for peripheral tissues, whereas butyrate plays a crucial role in providing energy to intestinal epithelial cells and stimulating cell growth and development (59). Previous studies indicated that ETEC infection may cause a decrease in VFAs in the intestinal tract of piglets (34, 37, 59). Consistent with these findings, in this study, ETEC infection (BD + ETEC) resulted in decreased concentrations of total VFA, acetic acid, propionic acid, butyric acid, valeric acid, and isovaleric acid. Berberine (LB + ETEC) notably elevated the concentrations of total VFA, acetic acid, propionic acid, valeric acid, and isovaleric acid in ETEC-challenged pigs (Table 2). This prompted a deeper exploration of the impact of berberine on the intestinal microbiota of ETEC-infected weaned piglets.

The establishment of the intestinal microbiota in children and piglets during infancy is a critical process. However, the microbial structure during this stage was unstable and vulnerable to environmental influences (35). ETEC infection can disrupt the intestinal microbiota, causing an imbalance that leads to detrimental effects, eventually resulting in diarrhea (6, 29). The alpha diversity (ACE richness index, Chao1 richness index, and Shannon diversity index) of the intestinal microbiota in the BD + ETEC group was significantly lower than that in the BD + Saline group (Figure 5A). Our findings are consistent with those of previous studies (28, 29, 35), indicating that ETEC infection notably diminishes the abundance and diversity of the piglet intestinal microbiota. The ACE and Chao1 richness indices in the LB + ETEC and HB + ETEC groups were significantly higher than those in the BD + Saline group. Additionally, the Shannon diversity index was higher, and the Simpson index was lower in the LB + ETEC group than in the BD + ETEC group (Figure 6A). These findings indicate that berberine increases the volume and variety of gut flora in piglets after weaning. Some studies have proposed that the similarity in the diversity pattern illustrates a comparable gastrointestinal health status among dual sets of hosts (29). Beta diversity analysis indicated a clear separation between the microbiota of the BD + ETEC and BD + Saline groups (Figures 5B, C), suggesting a significant effect of ETEC infection on the intestinal microbiota composition and structure of piglets. Furthermore, the microbiota of the LB + ETEC group differed distinctly from those of the BD + ETEC and HB + ETEC groups (Figures 6B, C). These findings, in conjunction with the results on the intestinal microbial structure, imply that prolonged berberine consumption by piglets induces substantial alterations in their intestinal microbiota composition and structure.

Moreover, the gut microflora significantly contributes to the maturation of intestinal functions and the overall health of both children and piglets (35). An imbalance in the intestinal microbiota is strongly linked to the occurrence of diarrhea (28). In our study, ETEC infection (BD + ETEC) decreased the relative abundances of *Parabacteroides, Barnesiella, Lactobacillus, Bacteroides, Lactococcus*, and *Holdemania* (Figure 5G). Studies have indicated that increased levels of *Parabacteroides* are linked to beneficial microbial communities and play a role in decreasing inflammation (60, 61). Intestinal *Barnesiella, Holdemania*, and *Bacteroides* produce VFAs that have anti-inflammatory effects and improve intestinal barrier function (62–64). A reduction in the presence of *Bacteroides* could be considered a key indicator of physiological diarrhea in post-weaning piglets (67). *Lactobacillus* is well-known for its production of lactic acid, which exhibits bactericidal properties. Moreover, the increased abundance of the genus *Lactobacillus* has been linked to enhanced mucus production, thereby improving intestinal barrier function and overall intestinal health (65). *Lactococcus* was the most abundant microbiota in the intestine. Research has demonstrated their impact on the evolution of the host gastrointestinal function, encompassing the enhancement of the immune response, digestion, microbiome establishment, and protection against harmful agents (66). The results indicated a significant correlation between beneficial bacteria and various physical, chemical, and immune barrier function marker genes in the small intestine of piglets. This suggests that a decrease in the abundance of these beneficial bacterial communities following ETEC infection could negatively affect the maintenance of intestinal mucosal barrier function in piglets. Additionally, after ETEC infection, the relative abundances of *Corynebacterium, Olsenella, Turicibacter, Enterobacter*, and *Cronobacter* significantly increased in the BD + ETEC group (Figure 5G). *Corynebacterium* is increasingly recognized as an important pathogen that can induce an inflammatory response (67). Similarly, growing evidence has demonstrated that *Olsenella* is commonly considered harmful and its increased abundance is closely linked to inflammation and impaired barrier function in piglets (68, 69). *Turicibacter* has also been identified as a pathogen that triggers inflammation (70). *Enterobacter*, a significant producer of LPS, has been linked to chronic inflammation in the intestine and body, potentially contributing to intestinal epithelial damage and metabolic disorders (71). Moreover, the integrity of the intestinal barrier is affected by the presence of *Cronobacter* (72). These results suggest that ETEC infection causes a decrease in VFA-producing bacteria, such as *Barnesiella, Holdemania*, and *Bacteroides*, as well as a reduction in beneficial bacterial microbiota that play a role in the physical (e.g., *Barnesiella, Lactobacillus, Bacteroides*, and *Lactococcus*), chemical (e.g., *Lactobacillus, Bacteroides*, and *Lactococcus*), and immune (e.g., *Parabacteroides, Bacteroides, Lactococcus*, and *Holdemania*) barriers of the small intestine in piglets. Moreover, there was a notable increase in potentially pathogenic and inflammation-inducing bacteria such as *Corynebacterium, Turicibacter, Enterobacter*, and *Cronobacter*. The disruption in the balance between beneficial and pathogenic bacteria caused by ETEC infection damages the intestinal barrier in piglets.

To investigate the impact of dietary Berberine on the intestinal microbiota of weaned piglets infected with ETEC, we analyzed notable alterations in microbiota abundance at the genus level within the Berberine-treated groups (LB + ETEC and HB + ETEC groups). Furthermore, we explored the correlation between these changes and the expression of marker genes associated with the small intestinal barrier function. Our study found that berberine supplementation significantly increased the relative abundances of *Enterococcus, Holdemanella, Weissella, Pediococcus, Muribaculum, Colidextribacter, Agathobacter, Roseburia, Clostridium, Fusicatenibacter*, and *Bifidobacterium* in the intestines of piglets (Figure 6G). *Enterococcus*, which is typically located in the gastrointestinal tract of humans and animals, has been used as a probiotic without any safety concerns (73). Studies have indicated that *Enterococcus* may aid in the management of inflammatory responses. Moreover, viral infections can reduce the abundance of *Enterococcus* in piglet intestines (74). Our study also found a significant correlation between *Enterococcus* and jejunal IL-1β and IL-8 and ileal MUC2 (Figures 7E, F). These findings suggest that an increased abundance of *Enterococcus* is beneficial for maintaining the immune barrier function of the jejunum and the chemical barrier function of the ileum in piglets. The genus *Holdemania* exhibits anti-inflammatory effects by releasing VFAs (62). Our study found a strong correlation between *Holdemania* abundance and CYP3A4 levels in the jejunum (Figure 7E), indicating that an increase in *Holdemania* abundance may play a role in maintaining chemical barrier function in the jejunum of piglets. *Weissella* has been recognized as a biomarker of healthy microbiota and has shown promise as a potential probiotic for promoting intestinal health (75). Our study revealed a significant correlation between *Weissella* and IL-1β and IL-6 in the duodenum, as well as IL-8 and BCL2 in the jejunum (Figures 7D, E). These findings indicate that an increase in the abundance of *Weissella* helps maintain immune barrier function in the duodenum and jejunum, while also inhibiting apoptosis in jejunal cells. *Pediococcus*, a probiotic, enhances the intestinal histological morphology and immune functions (76). Our study revealed a significant correlation between *Pediococcus* and various markers such as P-gly, CYP3A4, and CASP3 in the duodenum; IL-6 in the jejunum and ileum; and BCL2 in the ileum (Figures 7D–F). These results indicate that a higher abundance of *Pediococcus* may contribute to preserving the chemical barrier in the duodenum, enhancing the immune barrier in the jejunum, and suppressing apoptosis in the ileum. *Muribaculum* produces VFAs in the gut as metabolites with anti-inflammatory properties, which have been linked to longevity (77). Our findings suggested a significant correlation between *Muribaculum* and various markers in the duodenum and jejunum. In the duodenum, *Muribaculum* abundance positive correlated with Occludin, E-cad, P-gly, CYP3A4, IL-8, IFN-γ, BCL2, BAX, and BAK. In the jejunum, it positive correlated with E-cad, IL-8, IFN-γ, and CASP9 (Figures 7D, E). These results imply that an increase in *Muribaculum* abundance may help maintain the barrier functions (physical, chemical, and immune) of both the duodenum and jejunum and regulate cellular apoptosis. *Colidextribacter*, a potentially beneficial bacterium, may assist in regulating systemic inflammatory responses and preserving the integrity of the intestinal mucosa (78). A significant correlation was found between *Colidextribacter* and key markers such as ZO-1, MUC2, and BAX in the duodenum, and ZO-1, Occludin, MUC2, and P-gly in the jejunum (Figures 7D, E). This suggests that *Colidextribacter* plays a role in maintaining the physical and chemical barrier functions of both the duodenum and jejunum in piglets. *Agathobacter*, a VFA-producing bacterium, is considered a beneficial commensal and potential probiotic candidate because of its ability to produce butyric acid, which may have anti-inflammatory properties (79). Our findings showed a significant negative correlation between *Agathobacter* and levels of TNF-α in the duodenum, as well as IFN-γ and CASP9 in both the duodenum and jejunum (Figures 7D, E). This suggests that increasing the relative abundance of *Agathobacter* could potentially reduce inflammation in the duodenum and jejunum while also maintaining the immune barrier function of these regions in piglets. The genus *Roseburia*, which is known for its VFA production, plays a crucial role in preserving gut homeostasis and influencing immune development (80). Our research revealed a notable correlation between *Roseburia*, E-cadherin, and MUC2 in the ileum (Figure 7F), indicating that higher levels of *Roseburia* contribute to the maintenance of physical and chemical barrier functions in the ilea of piglets. *Clostridium* is considered beneficial because of its population of VFA-producing bacteria with anti-inflammatory properties that contribute to various benefits for intestinal homeostasis (81, 82). Our study aligns with previous findings, showing a significant correlation between *Clostridium* and E-cad, P-gly, IL-8, and TNF-α in the duodenum; ZO-1, Claudin-1, IL-1β, IL-6, IL-8, TNF-α, IFN-γ, BCL2, BAX, and CASP9 in the jejunum; and ZO-1, and CASP9 in the ileum (Figures 7D–F). This suggests that an increased abundance of *Clostridium* can help maintain the physical barrier function in the small intestine of piglets, maintain the immune barrier function of the duodenum and jejunum, and regulate cell apoptosis in the jejunum and ileum. *Fusicatenibacter* is a VFA-producing bacterium that suppresses intestinal inflammation (83). Our study revealed a significant correlation between *Fusicatenibacter* and CASP9 in the duodenum, BAK in the jejunum, as well as Claudin-1, MUC2, IL-1β, and BAK in the ileum (Figures 7D–F). This suggests that an increase in the abundance of *Fusicatenibacter* may play a role in regulating the apoptosis of piglet small intestine cells and preserving the physical, chemical, and immune barrier functions of the ileum. *Bifidobacterium* are crucial for preserving the integrity of the intestinal mucosal barrier through the production of VFAs and the maintenance of intestinal homeostasis (84). Our research revealed a notable correlation between *Bifidobacterium* and various markers such as ZO-2, E-cad, MUC2, BCL2, and CASP9 in the duodenum, as well as Occludin, MUC2, BCL2, and CASP9 in the jejunum, and IL-1β and IL-8 in the ileum (Figures 7D–F). This suggests that an increase in *Bifidobacterium* abundance contributes to maintaining the physical and chemical barrier functions of the duodenum and jejunum in piglets while also regulating the immune barrier function of the ileum and cell apoptosis in the duodenum and jejunum.

Our results also showed that dietary berberine significantly reduced the abundances of *Prevotella, Paraprevotella, Corynebacterium, Catenisphaera, Streptococcus, Enterobacter*, and *Collinsella* in the intestines of piglets (Figure 6G). *Prevotella* can promote inflammatory immune responses in the mucosa (68). Additionally, *Paraprevotella* is a significant pathogen within the intestinal environment and is involved in the development of various human diseases through its role in perpetuating chronic inflammation (85). Consistent with previous research (86), berberine significantly decreased the abundance of *Prevotella* and *Paraprevotella* and a correlation between *Prevotella* and P-gly and CYP3A4 in the duodenum and CASP9 in the jejunum was observed (Figures 7D, E). Additionally, the abundance of *Paraprevotella* was correlated with Claudin-1, E-cad, IL-6, IL-8, and IFN-γ in the duodenum, the ZO-1, IL-1β, IL-8, TNF-α, IFN-γ, BCL2, and BAX in the jejunum, as well as ZO-1 and CASP9 in the ileum (Figures 7D–F). Decreases in the abundance of *Prevotella* and *Paraprevotella* are associated with the maintenance of barrier functions in the duodenum and jejunum and the regulation of cell apoptosis in the jejunum and ileum. *Corynebacterium* is increasingly recognized as a pathogen capable of triggering inflammatory responses (67). Meanwhile, *Catenisphaera* has been identified as a producer of inflammatory cytokines (87). Our research revealed a notable association between *Corynebacterium* and IL-1β in the duodenum (Figure 7D). Significant correlations were observed between *Catenisphaera* and ZO-2, MUC2, and CASP9 in the duodenum as well as with ZO-1, IL-1β, and IL-8 in the ileum (Figures 7D, F). Consistent with previous studies, the marked reduction in the relative abundance of both *Corynebacterium* and *Catenisphaera* contributed to the maintenance of the physical and chemical barrier functions of the duodenum, thereby affecting the physical and immune barrier functions of the ileum. Microbial colonization of piglet intestines begins immediately after birth with initial colonization by *Streptococcus*, a common pathogenic bacterium (88). In the duodenum, *Streptococcus* significantly correlated with ZO-2, Occludin, MUC2, BCL2, BAX, BAK, and CASP9. In the jejunum, it was correlated with ZO-2, Occludin, E-cad, and MUC2, while in the ileum, it was correlated with IL-1β and IL-8 (Figures 7D–F). These findings suggest that reducing the relative abundance of *Streptococcus* could be beneficial for maintaining the barrier functions of the duodenum and jejunum as well as the immune barrier function of the ileum and for regulating duodenal cell apoptosis. *Enterobacter* induces chronic inflammation and damages intestinal barrier function (71). In our study, we observed correlations between *Enterobacter* with TNF-α and IFN-γ in the duodenum, MUC2 and IFN-γ in the jejunum, and Occludin and CYP3A4 in the ileum (Figures 7D–F). Therefore, reducing the relative abundance of *Enterobacter* may be beneficial for maintaining the immune barrier in the duodenum and jejunum of piglets, as well as for preserving the physical and chemical barrier function of the ileum. *Collinsella* is commonly described as a pathobiont linked to compromised intestinal barrier function (65, 89). Our study found a significant correlation between *Collinsella* and IL-6 in the duodenum as well as between Claudin-1 and E-cad in the ileum (Figures 7D, F). This suggests that reducing the abundance of *Collinsella* may have a positive impact on immune barrier function in the duodenum and physical barrier function in the ileum. Dietary berberine significantly increased the abundance of beneficial bacteria, including VFA-producing bacteria (e.g., *Muribaculum, Agathobacter, Roseburia, Clostridium, Fusicatenibacter*, and *Bifidobacterium*). It also increased the abundance of bacteria associated with maintaining intestinal physical barrier function (e.g., *Muribaculum, Colidextribacter, Roseburia, Clostridium, Fusicatenibacter*, and *Bifidobacterium*), those associated with maintaining intestinal chemical barrier (e.g., *Enterococcus, Holdemania, Pediococcus, Muribaculum, Colidextribacter, Roseburia, Fusicatenibacter*, and *Bifidobacterium*), and those associated with maintaining intestinal immune barrier (e.g., *Enterococcus, Weissella, Pediococcus, Muribaculum, Agathobacter, Clostridium, Fusicatenibacter*, and *Bifidobacterium*). In addition, berberine reduced the relative abundance of pathogenic and harmful bacteria, such as *Prevotella, Paraprevotella, Corynebacterium, Catenisphaera, Streptococcus, Enterobacter*, and *Collinsella* in the intestine. These changes in the intestinal microbiota help alleviate the damage to the piglet intestinal barrier function caused by ETEC infection.

## 5 Conclusions

This study elucidated the regulatory mechanisms of berberine in alleviating intestinal mucosal barrier injury induced by ETEC infection in a weaned piglet model. Berberine supplementation effectively mitigated the ETEC-induced intestinal mucosal barrier damage in weaned piglets. The beneficial effects of berberine included restoring the balance of the intestinal microbiota, increasing the presence of beneficial bacteria such as VFA-producing bacteria, and reducing the abundance of harmful and pathogenic bacteria. Furthermore, berberine increased the number of goblet cells and mucosal thickness in the small intestine, thereby improving its chemical barrier function. Berberine also upregulated the expression of tight junction and adhesion junction genes, strengthened the anti-apoptotic ability of small intestinal epithelial cells, and maintained the physical barrier function of the small intestine, ultimately enhancing intestinal integrity and reducing permeability. Additionally, berberine decreased the expression of intestinal inflammatory factors and alleviated ETEC-induced damage to the intestinal immune barrier. In summary, berberine played a preventive role against ETEC-induced intestinal mucosal barrier damage by improving the homeostasis of intestinal microbiota in piglets, enhancing the physical, chemical, and immune barrier functions, and ultimately mitigating the intestinal mucosal barrier damage induced by ETEC infection in piglets.

## Data Availability

The datasets presented in this study can be found in online repositories. The names of the repository/repositories and accession number(s) can be found below: https://www.ncbi.nlm.nih.gov/bioproject/1110050 and https://www.ncbi.nlm.nih.gov/, SRA 29048606-29048629.
