# Supplementary material for: Berberine alleviates enterotoxigenic Escherichia coli-induced intestinal mucosal barrier function damage in a piglet model by modulation of the intestinal microbiome
Source: Front Nutr. 2025 Jan 14;11:1494348. doi: 10.3389/fnut.2024.1494348 (PMC11772193; doi:10.3389/fnut.2024.1494348)
Supplement: Supplementary file 1 [file Table_1.docx]

Supplementary Material

Berberine alleviates enterotoxigenic *Escherichia coli*-induced intestinal mucosal barrier function damage in a piglet model by modulation of the intestinal microbiome

Min Du^1^, Xinran Liu^1^, Xu Ji^3^, Yue Wang^1^, Xiaodan Liu^1^, Chunfang Zhao^1,2^, Erhui Jin^1,2^, Youfang Gu^1,2^, Hongyu Wang^1^, Feng Zhang^1,2*^

^1^College of Animal Science, Anhui Science and Technology University, Chuzhou, China

^2^Anhui Province Key Laboratory of Animal Nutrition Regulation and Health, Chuzhou, China

^3^Anhui Province Key Laboratory of Livestock and Poultry Product Safety Engineering, Institute of Animal Science and Veterinary Medicine, Anhui Academy of Agricultural Sciences, Hefei, China

*** Correspondence:**Feng Zhang
zhangfeng@ahstu.edu.cn

# Supplementary Tables

**Table S1**. Ingredients composition and nutrient specification of basal diet

| Items (%) | Basal diet |
| --- | --- |
| Corn | 37.50 |
| Extruded corn | 15.00 |
| Soybean meal | 15.00 |
| Extruded soybean | 10.00 |
| Whey power | 10.00 |
| Vitamin and mineral premix* | 4.00 |
| Fish meal | 2.50 |
| Egg yolk powder | 2.00 |
| Sucrose | 2.00 |
| Soybean oil | 2.00 |
| Analyzed nutrient specification (%) | |
| Dry matter | 88.2 |
| Crude ash | 5.80 |
| Crude protein | 17.04 |
| Ether extract | 5.60 |
| Crude fiber | 2.80 |
| Ca | 0.54 |
| Total P | 0.54 |

*Provided per kilogram of diet: Zn (ZnSO_4_·H_2_O), 100 mg; Cu (CuSO_4_·5H_2_O), 125 mg; Mn (MnSO_4_·H_2_O), 60 mg; Fe (FeSO_4_·H_2_O), 120 mg; I (Ca(IO_3_)_2_, 0.6 mg; Se (Na_2_SeO_3_), 0.30 mg; vitamin A, 10000 IU; vitamin D3, 2500 IU, vitamin 35 IU; vitamin K3, 3.0 IU; Vitamin B5, 40 mg; nicotinic acid, 60 mg; folic acid, 1 mg; biotin, 0.2 mg; vitamin B6, 4.0 mg; vitamin B2, 7.5 mg; vitamin B1, 5.0 mg; vitamin B12, 0.08 mg.

**Table S2.** Sequences of genes-special primers used for RT-qPCR.

| Genes | Accession number | Primer sequences (5’-3’) |
| --- | --- | --- |
| MUC2 | NM_002457.5 | F: AGGACGACACCATCTACCTCAC  R: CGTGTGGTTCTGGAACTTACTG |
| P-glycoprotein | NM_001163060.1 | F: CAAACGGCCATTTCAGCTTCA  R: GTCACCTTGGTGGACAGCTT |
| CYP3A4 | NM_001195509.1 | F: CCAAGGGGACCGTGATGATG  R: ATGCAGTTGCGGGGTCCAGT |
| BCL2 | XM_005666256.3 | F: ACCCAGGTCTCTGATGAACTCTTCC  R: CACGAGTGGCTCCATCTCCTTATTG |
| BAX | XM_013998624.2 | F: GCTGACGGCAACTTCAACTG  R: GAAGGAAGTCCAGCGTCCAG |
| BAK | XM_021098603.1 | F: ATGACATCAACCGGCGATAC  R: TTGATGCCACTCTCGAACAG |
| CASP3 | NM_214131.1 | F: TGGGATTGAGACGGACAGTG  R: CGCTGCACAAAGTGACTGGA |
| CASP9 | XM_003127618.4 | F: ACTGCCAAGCAAATGGTCCAG  R: CCATCTGTGCCATAAACAGCC |
| ZO-1 | XM_021098896.1 | F: CCTGAGTTTGATAGTGGCGTTGA  R: AAATAGATTTCCTGCTCAATTCC |
| ZO-2 | NM_001206404.1 | F: GCAGAGACAACCCCCACTTT  R: CGTTAACCATGACCACCCGA |
| Claudin1 | NM_001244539.1 | F: AAGGACAAAACCGTGTGGGA  R: CTCTCCCCACATTCGAGATGATT |
| Occludin | XM_005672525.3 | F: ACCCAGCAACGACATA  R: TCACGATAACGAGCATA |
| E-cadherin | NM_001163060.1 | F: CAAACGGCCATTTCAGCTTCA  R: GTCACCTTGGTGGACAGCTT |
| IL-1β | NM_214055.1 | F: CCAGCCAGTCTTCATTGTTCAG  R: GCTGGATGCTCCCATTTCTC |
| IL-6 | NM_214399.1 | F: TCCTCGGCAAAATCTCTGCAA  R: ACAAGACCGGTGGTGATTCTC |
| IL-8 | NM_213867.1 | F: GCCTTCTTGGCAGTTTTCCTG  R: TGGAAAGGTGTGGAATGCGTA |
| TNF-α | NM_214022.1 | F: TTCTGCCTACTGCACTTCGAG  R: AGGGCATTGGCATACCCAC |
| IFN-γ | NM_213948.1 | F: CAGCTTTGCGTGACTTTGTG  R: GGTCCACCATTAGGTACATCTGA |
| β-actin | XM_021086047.1 | F: GGACTTCGAGCAGGAGATGG  R: GCACCGTGTTGGCGTAGAGG |
| GAPDH | NM_001206359.1 | F: TCGGAGTGAACGGATTTGGC  R: CACCCCATTTGATGTTGGCG |
